# Characteristic cerebral perfusion pattern in neuronal intranuclear inclusion disease

**DOI:** 10.3389/fnins.2022.1081383

**Published:** 2022-12-07

**Authors:** Hong-Fei Tai, Tian-Tian Hua, Zai-Qiang Zhang, Yun-Yun Duan, Zhi-Zheng Zhuo, An Wang, Yi Zhou, Shao-Cheng Liu, Shan Lv

**Affiliations:** ^1^Department of Neurology, Beijing Tiantan Hospital, Capital Medical University, Beijing, China; ^2^National Clinical Research Center for Neurological Diseases, Beijing, China; ^3^Department of Radiology, Beijing Tiantan Hospital, Capital Medical University, Beijing, China; ^4^Tiantan Image Research Center, National Clinical Research Center for Neurological Diseases, Beijing, China

**Keywords:** neuronal intranuclear inclusion disease, cerebral blood flow, arterial spin labeling, cortex, deep brain region

## Abstract

**Background:**

Neuronal intranuclear inclusion disease (NIID), which pathogenesis remains largely unclear, is a neurodegenerative disease caused by GGC repeat expansion in *NOTCH2NLC* gene. As case studies have reported dynamic cortical perfusion changes in NIID, this study aimed to explore the cerebral perfusion pattern in NIID patients.

**Materials and methods:**

A total of 38 NIID patients and 34 healthy controls (HCs) were recruited, and 2 NIID patients who had had episodic symptoms within 2 months were excluded. Data on demographic characteristics and clinical features were collected. All participants underwent three-dimensional pseudo-continuous arterial spin labeling perfusion magnetic resonance imaging (MRI) scanning. Voxel-based comparisons of cerebral blood flow (CBF) were conducted.

**Results:**

NIID patients showed decreased perfusion in the cortex but increased perfusion in the deep brain regions compared with HCs. The regions with significant hypoperfusion were distributed in the bilateral frontal, temporal, parietal, and occipital gyri, with the left frontal gyrus being the most prominent. The regions with significant hyperperfusion included the bilateral basal ganglia, midbrain, pons, para-hippocampal, and parts of the bilateral cerebellum, fusiform, lingual, rectus, orbital, and cingulum anterior gyri, which were adjacent to the midline (all FDR-corrected *p* <0.05). When comparing the mean CBF value of the whole brain, no significant differences were observed between NIID patients and HCs (28.81 ± 10.1 *vs.* 27.99 ± 5.68 ml/100 g*min, *p* = 0.666). Voxel-based analysis showed no significant difference in cerebral perfusion between NIID patients with and without episodic symptoms. The perfusion within the bilateral middle frontal and anterior cingulate gyri showed positive correlations with MMSE and MoCA scores using age, sex, and education as covariates (*p* <0.005 uncorrected).

**Conclusion:**

NIID patients exhibited characteristic cortical hypoperfusion and deep brain hyperperfusion. The perfusion in the bilateral frontal lobe and cingulate gyrus was correlated with the severity of cognitive dysfunction. Cerebral perfusion change may be involved in NIID pathophysiology and serve as a potential indicator for monitoring NIID severity and progression.

## Introduction

Neuronal intranuclear inclusion disease (NIID) is a progressive neurodegenerative disease caused by GGC repeat expansion in the 5′ untranslated region of *NOTCH2NLC* gene (Sone et al., 2016; Deng et al., 2019; Tian et al., 2019). The clinical spectrum of NIID is very broad and encompasses various chronic symptoms, such as cognitive impairment, parkinsonism, tremor, cerebellar ataxia, autonomic dysfunction, peripheral neuropathy, and myopathy, as well as a group of episodic symptoms, including encephalitic episodes, stroke-like episodes, disturbance of consciousness, episodic headache, and epileptic seizure (Sone et al., 2011, 2014, 2016; Yamada et al., 2017; Hayashi et al., 2020; Qin et al., 2020; Wang et al., 2020).

The pathogenesis of these symptoms in NIID remains largely unclear. Recent case studies have reported dynamic cerebral perfusion changes during episodic neurogenic symptoms attacks in NIID patients. Patients with encephalitic episodes, stroke-like episodes, and recurrent disturbance of consciousness show regional decreased cortical cerebral blood flow (CBF) in the hyperacute stage (within several hours after the episode onset) (Fujita et al., 2017), subsequently increased CBF in the acute stage (within several days to 3 weeks) (Fujita et al., 2017; Ataka et al., 2021), and reduced CBF in the chronic stage (2 months later) (Ataka et al., 2021). Another study has reported that most NIID cases had regionally decreased cortical CBF on single photon emission computed tomography. However, these studies have only provided a preliminary observation of the intracranial perfusion in a small number of NIID patients without any control groups or only described the perfusion changes in a specific period of episodic attacks.

Utilizing magnetically labeled blood plasma as an endogenous contrast media, arterial spin labeling (ASL) perfusion is a non-invasive, rapidly repeatable method for quantitatively measuring CBF. While ASL-MRI provides information comparable to fluorodeoxyglucose (FDG)-positron emission tomography (PET) (Detre et al., 2012), it does not involve exposure to radioactive tracers. This study aimed to explore the pattern of intracranial perfusion changes in NIID patients compared with healthy controls (HCs) using non-invasive three-dimensional ASL, including patients with and without paroxysmal symptoms.

## Materials and methods

### Participants

Initially, 38 NIID patients who met the following inclusion criteria were recruited: (1) patients clinically presenting with neurologic symptoms in accordance with NIID, including cognitive impairment, parkinsonism, tremor, autonomic dysfunction, peripheral neuropathy, myopathy, ataxia, and various episodic neurogenic symptoms; (2) skin biopsy showing eosinophilic hyaline intranuclear inclusions on hematoxylin and eosin (H&E) staining in adipocytes, fibroblasts, sweat gland cells, or other cells, which were anti-p62-positive on immunochemical staining; (3) the number of GGC repeats in the 5′-UTR of *NOTCH2NLC* gene larger than 60 (Tian et al., 2019). Two patients were excluded to eliminate the effect of episodic symptoms on intracranial perfusion in the acute stage, as they had suffered from one or more forms of episodic attacks within 2 months before enrollment, including stroke-like episodes, encephalitic episodes, and disturbances of consciousness. We also recruited 34 age-and sex-matched HCs without histories of neurological or neuropsychological diseases.

### Clinical assessment

Demographics and clinical characteristics of patients, including sex, age, education level, disease onset age, disease duration, clinical symptoms, and physical examinations, were collected. Cognitive function was evaluated by mini-mental state examination (MMSE) and Montreal Cognitive Assessment (MoCA). According to whether patients had ever suffered from episodic symptoms since disease onset, NIID patients were divided into NIID with and without episodic symptoms.

### Magnetic resonance imaging acquisition

Magnetic resonance imaging (MRI), including 3D T1 weighted imaging (T1WI) and ASL, was performed using a 3.0 Tesla MR scanner (Philips Ingenia CX, Best, Netherlands). Sagittal 3D T1W images were acquired using magnetization-prepared rapid gradient echo (MPRAGE) (TR/TE = 6.6 ms/3 ms, TI = 880 ms, FA = 8°, image resolution = 1 mm × 1 mm × 1 mm, and slice number = 196). ASL images were obtained using 3D axial acquisition with gradient and spin echo (GRASE) (TR/TE = 4,700 ms/11 ms, FA = 90°, voxel size = 2 mm × 2 mm, slice thickness = 4 mm, matrix size = 128 × 128, slice number = 30, labeling duration = 2 s, and post-delay = 1.8 s). Other conventional sequences (T2WI, FLAIR, DWI, and T2*WI) were also performed to exclude major neurological disorders, such as tumors or stroke.

### Data processing and analysis

Cerebral blood flow (CBF) maps were generated with the software provided by the scanner vendor. Imaging data were preprocessed using Statistical Parametric Mapping 12 (SPM12^1^) and custom scripts on *MATLAB* software (*MATLAB* 2019b) with the following steps: (1) co-registration of the M0 image (obtained with the pCASL acquisition and the same image space with CBF) with the anatomical T1W image; (2) normalization of T1W images to MNI template using Segment in SPM; (3) CBF image warped into the MNI space using the forward transformation matrix derived from T1W segmentation. For whole brain level comparisons, the mean CBF within the whole brain was calculated by home-developed Matlab scripts.

### Statistical analysis

Using the SPSS 25.0 statistical package, demographic and clinical data of patients in different groups were compared using the χ^2^ test or Fisher’s exact test for categorical variables and two-sample *t*-test or Mann-Whitney *U*-test for continuous variables. A *p*-value of <0.05 (two-sided) was considered statistically significant.

Voxel-based analyses of CBF within brain tissue were performed using SPM, including two-sample *t*-tests between the two groups, analysis of covariance (ANCOA) of the subgroups, and the correlation between CBF and clinical variables were analyzed using linear regression, considering sex, age, and education level as covariates. Multiple comparisons were corrected with a false discovery rate (FDR) at *p* <0.05. If significant clusters were not found, a more liberal voxel-wise threshold at uncorrected *p* <0.005 with a cluster size of 50 voxels was adopted.

## Results

A total of 36 patients with NIID and 34 age- and sex-matched HCs were included in the analysis. The demographic and clinical features of the participants are summarized in Table 1. There was no significant difference between the two groups in age, sex, and education years (all *p* values >0.05). Significantly lower scores of MMSE and MoCA were found in the NIID group than that in the HC group (both *p* values <0.001).

**TABLE 1:** Clinical characteristics and cerebral blood flow (CBF) values of the subjects.

Variables	HC (*n* = 34)	NIID (*n* = 36)	*P*-value
Age (years)	60.94 ± 7.15	59.94 ± 9.88	0.632
Sex (male, %)	14 (41.2%)	19 (52.8%)	0.331
Education (years)	10.61 ± 4.02	11.6 ± 4.13	0.386
MMSE (scores)	29.30 ± 0.98	24.41 ± 5.34	<0.001
MoCA (scores)	27.25 ± 2.47	19.12 ± 6.61	<0.001

NIID, neuronal intranuclear inclusion disease; HC, healthy control; MMSE, mini-mental state examination; MoCA, montreal cognitive assessment.

### Comparison of cerebral perfusion between neuronal intranuclear inclusion disease and healthy controls

**FIGURE 1:**
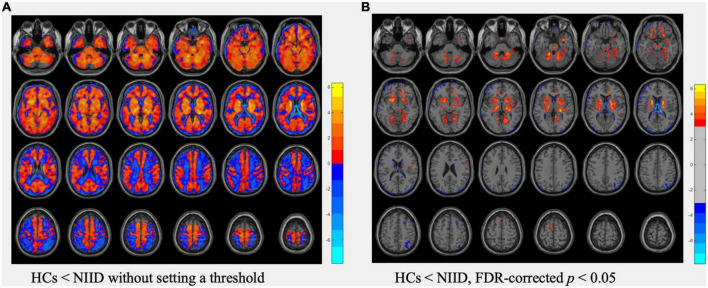
Comparisons of cerebral blood flow (CBF) data between healthy controls (HCs) and neuronal intranuclear inclusion disease (NIID) patients using *t*-test. **(A)** The T map of CBF differences without setting a threshold. **(B)** The T map thresholded using FDR-corrected *p* <0.05, showed regions with significant CBF differences between HCs and NIID. Color bars denote the *t* values.

In voxel-based analysis, the T map (Figure 1A) without setting a threshold qualitatively revealed an extensive area of decreased perfusion in the cortex but increased perfusion in the deep brain regions in NIID patients compared with HCs. Additionally, there was a gradually decreasing perfusion gradient trend from the deep region to the cortex. After setting the threshold at an FDR-corrected *p* <0.05, the regions with significant hypoperfusion were distributed in the bilateral frontal, temporal, parietal, and occipital gyri, with the left frontal gyrus being the most prominent. Furthermore, the regions with significant hyperperfusion included the bilateral basal ganglia, midbrain, pons, para-hippocampal gyrus, and parts of the bilateral cerebellum, fusiform, lingual, rectus, orbital, and cingulum anterior gyri, which were adjacent to the midline (Figure 1B).

When comparing the mean CBF value of the whole brain, no significant differences were observed between patients with NIID and HCs (28.81 ± 10.1 *vs.* 27.99 ± 5.68 ml/100 g*min, *p* = 0.666).

### Comparisons of cerebral perfusion between neuronal intranuclear inclusion disease patients with and without episodic symptoms

A similar pattern of perfusion change was found in the comparisons of HCs *vs.* NIID patients with episodic symptoms (*n* = 14) and HCs *vs.* NIID patients without episodic symptoms (*n* = 22, both *p* <0.05 FDR-corrected). Details regarding anatomical regions for each contrast are shown in Supplementary Figure. However, there were no significant differences in perfusion of any region between NIID patients with and without episodic symptoms (the clinical characteristics of the two groups are shown in Supplementary Table).

### Voxel-wise correlations between cerebral blood flow and clinical variables in neuronal intranuclear inclusion disease

**FIGURE 2:**
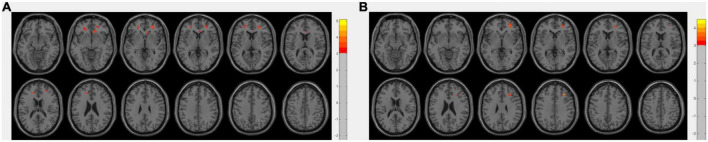
Areas with a positive correlation between perfusion level and mini-mental state examination (MMSE) score **(A)**, or montreal cognitive assessment (MoCA) score **(B)**, using linear regression adjusting for gender, age, and education, with a threshold at uncorrected *p* <0.005 with a cluster size of 50 voxels. Color bars denote the regression coefficient *t* value.

Abnormal perfusion within the bilateral middle frontal and anterior cingulate gyri showed positive correlations with cognitive function determined by MMSE and MoCA scores using age, sex, and education as covariates (*p* <0.005 uncorrected, Figure 2). Additionally, the perfusion showed no significant correlation with disease duration.

## Discussion

GGC repeat expansion in the 5′-untranslated region (5′-UTR) of *NOTCH2NLC* gene has been recognized as the causative mutation of NIID (Deng et al., 2019; Sone et al., 2019; Tian et al., 2019); however, NIID pathogenesis remains largely unclear. In the present study, we identified a characteristic pattern of cerebral perfusion change in NIID patients, showing hypoperfusion in the cortex and unexpected hyperperfusion in the deep brain regions. Previously, one study has observed cerebral perfusion in a group of NIID patients using single photon emission computed tomography, and the results showed that most patients (27/29) had regional hypoperfusion in the cortex (Sone et al., 2016). The present study clarified that the cerebral cortex perfusion in NIID patients was generally reduced, with frontal lobes being the most prominent. Additionally, the perfusion of bilateral frontal lobes and cingulate gyrus was related to the cognitive function of NIID patients. These findings suggested that vascular perfusion mechanisms were involved in NIID pathophysiology, and cerebral perfusion was a potential biomarker for monitoring NIID severity and progression.

We found that deep brain regions in NIID patients were excessively hyperperfused rather than reduced. When comparing the mean CBF value of the whole brain between NIID patients and HCs, the reverse perfusion change in the cortex and deep brain regions could be offset by each other. Interestingly, the imaging changes of the two areas on conventional head MRI of NIID patients are also different. The cerebral cortex with decreased perfusion on ASL manifests as chronic cortical atrophy, while the deep brain regions with hyperperfusion on ASL typically exhibit extensive white matter T2/Flair hyperintensity, which might be related to that hyperperfusion state bringing about a blood-brain barrier breakthrough and consequentially extravasation of fluid and vasogenic edema (McKinney et al., 2007).

The dynamic brain perfusion changes during episodic neurogenic symptoms in NIID patients, showing decreased CBF in focal cortical regions in the hyperacute stage and subsequent hyperperfusion lasting for days to several weeks (Fujita et al., 2017; Ataka et al., 2021), are similar to that in mitochondrial encephalomyopathy, lactic acidosis, and stroke-like episodes (MELAS) syndrome (Li et al., 2019). Recent studies have found mitochondrial swelling and downregulated mitochondrial oxidative phosphorylation in NIID muscle biopsies (Yu et al., 2022). There may also be possible shared mechanisms underlying both diseases. Rodan et al. (2015) have demonstrated that patients with MELAS between stroke-like episodes also showed cortical hyperperfusion and inversely proportional reduction in cerebral vascular reactivity (CVR), which was considered to be the result of an adaptive response in an attempt to compensate for the metabolic imbalance due to inefficient ATP generation from oxidative metabolism by abnormal mitochondria. Similarly, the present study revealed that NIID patients had cerebral hyperperfusion in the deep regions of the brain, while neuropathological evidence shows that the perivascular areas are relatively preserved even in the context of severe white matter damage (Yoshii et al., 2022). We speculate that hyperperfusion may also result from compensatory vasodilation to compensate for energetic metabolism disturbance caused by mitochondrial disorders in NIID, which is yet to be fully elucidated in future studies.

Taken as a whole, cerebral perfusion in NIID patients exhibits a high-to-low gradient from the deep brain to the cortex. This characteristic perfusion pattern probably might result from the combination of multiple factors. First, the perfusion gradient was exactly consistent with the pressure gradient across cerebral arteries, with a marked drop in blood pressure from large and medium-sized cerebral vessels to cerebral peripheral beds (Blanco et al., 2017). At the base of the brain (the “vascular centrencephalon”), short straight arteries transmit blood pressure directly from large arteries to small resistance vessels; however, the cerebral convexity is supplied by long arteries with many branches, resulting in a drop in blood pressure. Second, autonomic dysfunction is very common in NIID, leading to reduced cerebrovascular activity and vascular dysregulation, and the pressure/perfusion gradient is further amplified in NIID patients. While the deep brain regions exhibit chronic compensatory hyperperfusion, the areas near the cortex tend to show decompensated hypoperfusion. In conclusion, chronic hypoperfusion in the cortex, compensatory hyperperfusion in the deep brain regions, and vascular dysregulation may contribute to NIID pathophysiology cooperatively.

Despite the promising results, the limitations of this study should be considered. First, this study included 14 NIID patients who had ever had episodic attacks. Although the interval between the last episode and the MRI scan was more than 2 months, some patients might continue to have focal cortical hyperperfusion after the episode for a long time or develop excessive hypoperfusion in the focal cortex due to focal brain atrophy. As we have compared the cerebral perfusion between NIID patients with and without episodic attacks without finding a significant difference, the impact on study results was likely to be negligible. Second, the clinical manifestations of NIID are highly heterogeneous, and there is a lack of a verified scale for evaluating the severity of the disease, which makes it impossible to accurately evaluate the correlation between NIID severity and cerebral perfusion. Here, we only analyzed the correlation between cognitive function and perfusion in NIID. Third, as a preliminary study, we observed the cerebral perfusion change of NIID using ASL, and more data were required to further confirm our findings. In addition, it is necessary to evaluate cerebrovascular reactivity and autoregulative capacity in future studies to better clarify the underlying pathophysiological mechanisms.

## Data availability statement

The raw data supporting the conclusions of this article will be made available by the authors, without undue reservation.

## Ethics statement

The studies involving human participants were reviewed and approved by Medical Ethics Committee of Beijing Tiantan Hospital, Capital Medical University. The patients/participants provided their written informed consent to participate in this study.

## Author contributions

Z-QZ and H-FT: study concept, design, and drafting of the manuscript. H-FT, T-TH, Y-YD, AW, Z-ZZ, YZ, and SL: acquisition of data. H-FT, T-TH, Z-QZ, Y-YD, and Z-ZZ: analysis and interpretation of the data. Z-QZ and Y-YD: study supervision. Z-QZ: critical revision of the manuscript. All authors contributed to the article and approved the submitted version.

## Footnotes

1
http://www.fil.ion.ucl.ac.uk/spm
