# Supplementary material for: Characteristic cerebral perfusion pattern in neuronal intranuclear inclusion disease
Source: Front Neurosci. 2022 Dec 7;16:1081383. doi: 10.3389/fnins.2022.1081383 (PMC9768440; doi:10.3389/fnins.2022.1081383)
Supplement: Supplementary file 1 [file Data_Sheet_1.docx]

Supplementary Material

## Supplementary Figure


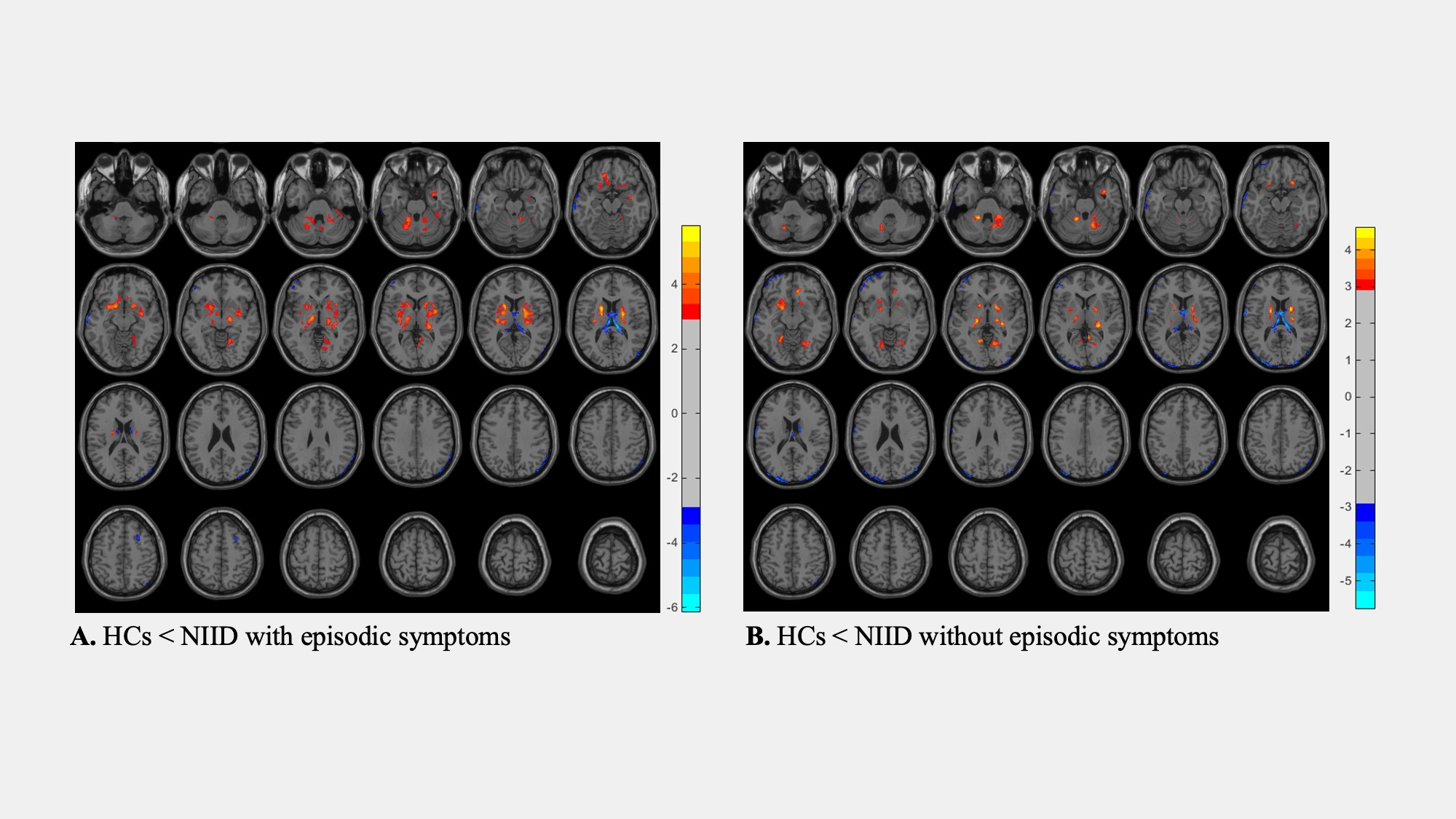


**Supplementary Figure.** Comparisons of CBF data. (A) CBF differences between NIID patients who had ever had episodic symptoms more than 2 months before and HCs. (B) CBF differences between NIID patients without any episodic symptoms and HCs (FDR-corrected *p* < 0.05).

**Supplementary Table. Clinical characteristics and CBF values between NIID patients without episodic symptoms and the patients with episodic symptoms more than two months before.**

| Variables | NIID with episodic symptoms more than two months before  (n=14) | NIID without episodic symptoms  (n=22) | *p*-value |
| --- | --- | --- | --- |
| Age (years) | 56.86 ± 13.75 | 61.91 ± 5.94 | 0.137 |
| Sex (male, %) | 6 (42.9%) | 13 (59.1%) | 0.342 |
| Education (years) | 10.29 ± 4.46 | 10.82 ± 3.80 | 0.704 |
| Duration (years) | 5 (2, 11) | 5 (2.75, 8) | 0.707 |
| MMSE (scores) | 25.08 ± 4.64 | 24.04 ± 5.76 | 0.596 |
| MoCA (scores) | 18.50 ± 5.36 | 19.45 ± 7.29 | 0.694 |
| Whole brain CBF (ml/100 g*min) | 29.85 ± 9.18 | 28.14 ± 10.80 | 0.628 |

NIID, neuronal intranuclear inclusion disease; HC, healthy control; MMSE, Mini-mental State Examination; MoCA, Montreal Cognitive Assessment; CBF, cerebral blood flow.
